# Genome Scale Transcriptomics of Baculovirus-Insect Interactions

**DOI:** 10.3390/v5112721

**Published:** 2013-11-12

**Authors:** Quan Nguyen, Lars K. Nielsen, Steven Reid

**Affiliations:** Australian Institute for Bioengineering and Nanotechnology, The University of Queensland, St Lucia, QLD 4072, Australia; E-Mails: quan.nguyen@uq.edu.au (Q.N.); lars.nielsen@uq.edu.au (L.K.N.)

**Keywords:** baculovirus, insect virus, virus-host interactions, microarray, RNA sequencing, next generation sequencing, genome scale, expression profile

## Abstract

Baculovirus-insect cell technologies are applied in the production of complex proteins, veterinary and human vaccines, gene delivery vectors‚ and biopesticides. Better understanding of how baculoviruses and insect cells interact would facilitate baculovirus-based production. While complete genomic sequences are available for over 58 baculovirus species, little insect genomic information is known. The release of the *Bombyx mori* and *Plutella xylostella* genomes, the accumulation of EST sequences for several Lepidopteran species, and especially the availability of two genome-scale analysis tools, namely oligonucleotide microarrays and next generation sequencing (NGS), have facilitated expression studies to generate a rich picture of insect gene responses to baculovirus infections. This review presents current knowledge on the interaction dynamics of the baculovirus-insect system‚ which is relatively well studied in relation to nucleocapsid transportation, apoptosis, and heat shock responses, but is still poorly understood regarding responses involved in pro-survival pathways, DNA damage pathways, protein degradation, translation, signaling pathways, RNAi pathways, and importantly metabolic pathways for energy, nucleotide and amino acid production. We discuss how the two genome-scale transcriptomic tools can be applied for studying such pathways and suggest that proteomics and metabolomics can produce complementary findings to transcriptomic studies.

## 1. Introduction

Baculoviruses are enveloped viruses that contain a circular double stranded DNA genome (80–180 kb) [1]. Baculoviruses are specifically infectious to invertebrates, most commonly insect species belonging to the Lepidoptera order, and there is no evidence of cross-order transmission [2]. Baculoviruses can also enter a range of mammalian cell types including human, primates, rodent, rabbit, porcine, bovine, and other animals such as fish and avian species, but neither replicate nor transpose baculovirus DNA into host chromosomes in these cell types [3,4]. Hence, the viruses are safe to vertebrates and have been approved by the US Food and Drug Administration and European Medicine Agency for manufacturing of veterinary and human vaccines [5]. 

Over 40 years of research on molecular biology of baculoviruses has produced a rich knowledge about baculovirus gene expression profiles and gene functions during infection, including: virus entry, movement into the cell nucleus, DNA unpackaging, early transcription, DNA replication, late transcription, translation, budded virus (BV) assembly, BV budding, occlusion derived virus (ODV) assembly, occlusion, and release of occlusion bodies (for an overview of baculovirus molecular biology studies, refer to Rohrmann [6]). However, little is known about host responses towards baculovirus infections.

Baculoviruses exert vigorous effects on host cells. Within a short infection time frame, the viruses rapidly propagate and change host cells by arresting host cell cycle progression, creating a “viral pseudo S phase”, remodeling the cellular cytoskeleton, forming virogenic stroma in the nucleus as a specialized compartment for replication, undertaking transcription and packaging, and eventually breaking-down the host nuclei. In the *Helicoverpa armigera* nucleopolyhedrovirus (HearNPV)*-H. zea* system, within 48 hours post infection (h.p.i), baculoviruses can produce over 250,000 genomes per cell, which is 20 times more DNA than that produced in an uninfected host cell, and the total viral mRNA from 134 virus genes is higher than the total cellular mRNA produced from over 15,000 genes during normal cell growth [7]. Similarly, in the *Autographa californica* multicapsid nucleopolyhedrovirus (AcMNPV) infected *Spodoptera frugiperda* cell line (Sf9) and AcMNPV infected *Trichoplusia ni* cell line (Tnms42), as high as one million viral genomes per haploid host insect genome was reported [8]. 

Although infected cells are controlled by baculoviruses, and a global reduction of host proteins and mRNAs occurs, host cells also respond by up-regulating or repressing certain genes and cellular pathways. A number of genes escape from the global expression shutoff and several host cellular pathways such as DNA damage responses, heat shock responses, pro-survival responses, apoptosis, energy metabolism, iron-ion transport, ubiquitin-proteasomal degradation and miRNA pathways have been shown to be up-regulated following infections by recent studies [9,10,11,12,13]. 

Transcriptomics is a powerful tool to study baculovirus-insect systems at the mRNA level, which is arguably the most important level of regulation during a baculovirus infection. Previous studies by Iwanaga *et al.* [14] using BmNPV infected cells suggested that infection events in the host cell were strongly regulated at the mRNA expression level rather than at the translational level. Gatehouse *et al.* [15] proposed that protein and RNA degradation are the key processes for controlling the global shutoff pattern induced by a baculovirus infection. In addition, polysome profiles, which were mRNA-ribosome complexes extracted by sucrose gradient centrifugation of cytosolic fractions of infected cells, were similar between Sf9 cells with and without an AcMNPV infection, indicating that active translation activities take place in infected cells even at late infection stages (16 h.p.i) [16]. Usually, it was found that changes in protein levels were due to prior changes in mRNA levels. Huynh *et al.* [17] showed that for high cell density infections in cultures, a reduction in the expression of the β-galactosidase protein in Sf9 cells infected by a recombinant AcMNPV virus was due to early infection events, most likely leading to a decrease in mRNA levels following a reduction in virion genome numbers.

To study host responses, the lack of insect genome sequences is a challenge. To overcome this challenge, rapid developments of RNA sequencing (RNA-seq) technologies for generating genome-scale transcript sequences in non-model organisms can be utilized [18,19]. This review shows that most of our understanding about insect host responses to baculovirus infections so far has been derived from microarray and RNA-seq studies in the last five years. These studies, however, investigated different host-baculovirus systems, under different infection conditions, and hence identified different sets of regulated genes. This review identifies possible conservative changes and knowledge gaps relating to host-baculovirus interactions, and proposes the further use of transcriptomics to investigate genome-scale interactions, with more focus on host responses. Finally‚ the review briefly discusses challenges and opportunities for genetic engineering of host genes, determined by transcriptomic studies, to moderate the infection process in order to increase product yields using insect cell technology, which is important for commercialization of the technology. For expression of some recombinant proteins, efficacy remains the key challenge in relation to commercial production but for many protein products and certainly for wild type baculoviruses to be used as biopesticides, yield improvements are essential for insect cell technology to be competitive in relation to alternative manufacturing systems. 

## 2. Host Genes/Pathways that Baculoviruses Require for Successful Infection Processes in Insect Cells

### 2.1. Transcriptomic Studies

Transcriptomic studies of baculovirus gene expression have expanded current understanding extensively. One of the first microarray studies for baculovirus genes was by Yamagishi *et al.* [20], in which PCR amplified fragments of virus genes were printed on 192 spots of a microarray chip and used as probes for hybridization. The study found four virus genes, namely p10, p35, lef-3 and lef-6, were involved in differential responses of host cells to AcMNPV infection, which would lead to different productivities by two insect cell lines Sf9 and High-Five. Also, by applying DNA microarrays with cDNA probes from PCR, Jiang *et al.* [21] characterized sequential expression patterns of all 155 AcMNPV genes and found 12 virus genes that depended on the virus pe38 gene for expression. Recently, the use of NGS for analysis of baculovirus gene expression patterns produced unprecedented genome-scale analysis of baculovirus infection processes, including mapping of all transcription start sites and polyadenylation sites, splicing variants, and putative interactions of virus proteins expressed at certain infection phases with host proteins [8,11]. A recent study using NGS for differential gene expression analysis and bioinformatic prediction of protein-protein interaction network suggested 22 viral proteins that could interact with 2,326 host proteins, totaling almost 8,907 interactions [11]. Another RNA-seq study comprehensively characterized 218 transcription start sites and 120 polyadenylation sites of all AcMNPV virus genes [8]. The authors also detected the possible encapsidation of viral mRNAs for late genes, suggesting a novel mechanism that the virus potentially uses to establish early infection, which is currently believed to depend entirely on the host RNA polymerase. Their study also suggested that 12 virus genes carry splicing variants, rather than only one gene as previously thought. The study also found an unexpected scale of antisense transcription for 50 virus genes. Such genome-scale transcriptomic studies offer great capacity to answer many unanswered questions on baculovirus gene regulation. Table 1 outlines the current knowledge on functions of a number of baculovirus genes that interact with host genes, which have been identified, mainly by molecular experiments, to interact with insect hosts.

For transcriptomic studies in an insect host, genome-scale sequences of coding genes are valuable resources for studying insect responses to baculovirus infections. With international efforts, only two Lepidopteran genomes are available to date, namely the *B. mori* genome [22] and the recently sequenced Diamondback moth (*Plutella xylostella*) genome [23]. To overcome challenges due to the lack of genomic sequences, several research groups have applied the traditional Sanger sequencing method to different collections of expressed sequence tag (EST) libraries such as those from the fall armyworm *S. frugiperda* [24,25], or from *H. virescens* [26], but these efforts could generate sequences for only a portion of the genome, usually fewer than 5,000 coding genes. Recently‚ more powerful RNA-sequencing technology has revolutionized the exome sequencing of a variety of non-model organisms, including insect species such as *Manduca sexta* [27], *H. virescens* [28], *Galleria mellonella* [29], *L. dispar* [30], and *H. zea* [18]. These sequence resources have been contributing to the expression analysis of baculovirus-insect interactions.

Expression analysis using microarrays have been applied to different baculovirus infection setups to compare different virus-host interactions, including: permissive *vs.* non-permissive hosts [31], infection of fat body *vs.* haemocyte [31], infection of high-yield *vs.* low-yield cell lines [20], uninfected *vs.* infected cells [15,18,32,33] or to compare infections at different time-points [7,10]. RNA-sequencing experiments are also useful for the analysis of RNA differential expression of all insect genes and of non-coding RNA [8,11,19]. Reviewing findings by these studies may show common genes and pathways being regulated by baculovirus infections (Table 2).

**Figure 1:**
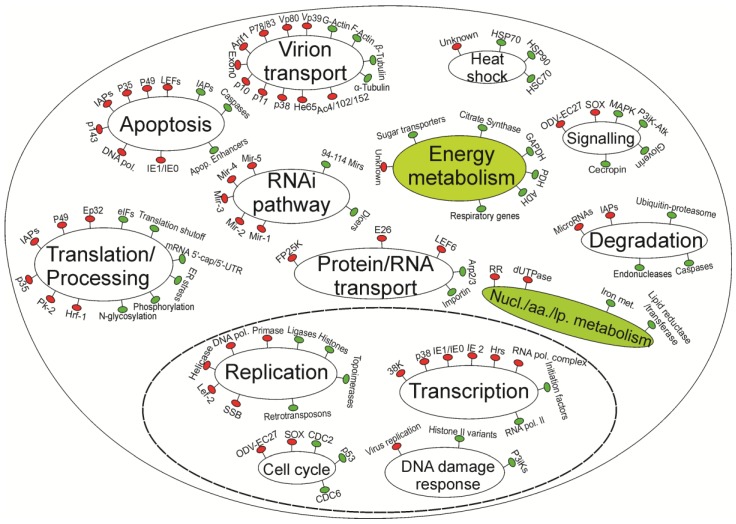
A simplified model of current understanding of host-baculovirus interactions. Virus genes/processes are in red, and insect genes/processes are in green. Major pathways are indicated by ellipses. The left half of the diagram represents pathways that are relatively well understood. The right half shows pathways that deserve more research, especially those indicated by filled ellipses. The ellipse with dashed boundary represents the nuclei. The nucl./aa./lp. abbreviation stands for nucleotide/amino acid/lipid metabolism processes.

The following subsections review in more detail the virus-insect interactions presented in Table 1, Table 2, and summarized in Figure 1 according to major pathways and cellular processes.

**Table 1:** Virus genes with known interactions with host genes or known effects on host functions.

Functional groups	Baculovirus genes/pathways	Baculovirus strains [6]	Functions	Ref.
Virus genes interacting with host cell receptors	GP-64, F protein	GP-64 only in group I alpha NPV‚ while the F protein in group II alpha-, beta- and delta- NPV	Virus-cell receptor attachment, facilitate entry by clathrin-mediated endocytosis processes	[34,35]
	Per OS infectivity factors (Pif-1, 2, 3, p-74)‚ and possibly Pif-4 and 5	All 5 Pifs and p-74 are core baculorius genes, and found in other invertebrate DNA viruses that replicate in the nucleus	Pif-1, 2, and 3 and p74 form a complex and facilitate ODV binding to midgut epithelial cell receptors	[36]
Apoptosis	IAP-1, IAP-2, IAP-3, IAP-4, and IAP-5	IAP1-4 in both NPVs and Granulosis viruses (GVs), while IAP-5 in GVs only. Each baculovirus strain has several/not all IAPs. IAP orthologs found in a number of hosts	Baculovirus IAPs mediate protein-protein interactions to block selected caspases. The IAP RING domain functions as an E3 ubiquitin ligase to trigger proteasome degradation of targeted caspases	[37]
	P35	AcMPNV, BmNPV, Culex nigripalpus NPV, Leucania separata MNPV, Maruca vitrata MNPV, T. ni MNPV and Clostera anachoreta	Binds to and inactivates host’s effector caspases	[38]
	p49	SpltMNPV, LsMNPV, SlNPV, AcMNPV, and HearNPV	Inhibits host’s initiator caspases (upstream of p35 but downstream of IAPs) and several host’s effector caspases	[39]
	Replicative lefs (lef-1, 2, 3, and 11) p-143, DNA pol and IE1/IE0	Lef-3 in Lepidopteran NPV and GV; Lef-1, Lef-2, Lef-11, and p-143 in all baculoviruses (except for Lef-11 not in CuniNPV), IE1/IE0 in all group I and II alpha baculoviruses	Trigger host DNA damage response and induce apoptosis	[40]
Cell cycle	ODV-EC27 (A virus multifunctional Cyclin)	In all baculoviruses	Interacts with host’s cdc-2 for cell cycle arrest at G2/M phase, or with host’s cdc-6 to override host check-point to allow DNA replication	[41]
	P33-sulfhydryloxidase (SOX)	In all baculoviruses	Forms stable complex with host’s p53 protein, preventing p53-induced apoptosis	[42]
Cytoskeleton and nucleocapsid transport	Protein kinase-1, Protein kinase-2	PK-1 found in Lepidopteran NPVs, and GVs, similar to some insect PK; PK-2 found in AcMNPV, BmNPV, PlxyNPV and RoMNPV	Actin cytoskeleton remodeling (protein-protein interaction prediction)	[11]
	Arif-1	All group I and most group II Alpha baculoviruses	Accumulates F-actin at the plasma membrane	[43]
	VP80 (a Paramyosin-like protein)/P78-83/VP39	VP80 and p78/83 in all group I and II Lepidopteran NPVs; VP39 in all baculovirus genomes	Interact with host’s F-actin filaments to transport nucleocapsids in the cytoplasm	[44,45]
	VP80	Vp80 found in all group I and II Lepidopteran NPVs	Interact with myosin motor proteins and F-actin to transport nucleocapsids to the nucleus periphery	[44]
	IE-1, PE38, HE65, Ac004, Ac102, Ac152	IE1 all baculoviruses; PE38 in all Group I NPV and four GV genomes	Accumulate host’s monomeric G-Actin into nucleus	[46]
	P78/83 (N-WASP-homologous protein) and ODV-C42	p78/83 all group I and II Lepidopteran NPVs	At early infection, transports nucleocapsid into nucleus by activating nuclear actin polymerization via an actin related protein (Arp2/3) complex. At late infection, facilitates actin assembly to form F-filament inside nucleus	[47,48]
	EXON0	In all Lepidopteran NPVs	Interacts with β-tubulin to facilitate binding of nucleocapsids to microtubules	[49]
	P10	In all group I and II NPVs and most GVs	Interacts with α-tubulin and mediates nuclear disintegration and cell lysis	[50]
Nucleo-cytoplasmic transport of viral proteins	FP25K and E26	FP25K in all Lepidopteran NPVs and GVs. E26 in group I Lepidopteran NPV	Together with host Importin-α-16, transport viral proteins into the inner nuclear membrane (INM)	[51]
Metabolism	ADP ribose pyrophosphatase (Ac38)	All Lepidopteran NPVs and GVs	The enzyme hydrolyzes ADP-ribose, an intermediate of metabolism of NAD+, mono- or poly-ADP-ribosylated proteins and cyclic ADP-ribose, thereby conferring detoxification effects	[52]
	P33-sulfhydryl oxidase (SOX)	In all baculoviruses	Flavin adenine dinucleotide (FAD)-binding sulfhydryl oxidase can play roles in protein disulphide bond formation and protection from oxidative stress	[53,54]
	Super oxide dismutase (SOD)	In most Lepidopteran baculoviruses	Converts superoxide into Hydrogen peroxide (possibly active in BmNPV, but this activity is not confirmed in AcMNPV)	[55,56]
Replication	Ribonucleotide reductase	Three GVs, 10 NPVs group II, OpMNPV and LdMNPV	Catalysis of ribonucleotides to deoxyribonucleotides for DNA synthesis	[57,58]
	DNA polymerase complex (Dnapol, helicase, primase, SSB, and LEF-2)	All baculoviruses	May require host’s DNA topoimerases and DNA ligases	[6,59]
	dUTPase	In nine group II NPVs, OpMNPV, and two GV genomes	Prevents incorporation of dUTP into DNA	[58,60]
Transcription	IE1/IE0, IE2, hrs, ADPRase (ADP-ribose pyrophosphatase)	IE1/IE0, hrs and ADPRase in all baculoviruses. IE2 in all Group I Lepidopteran NPVs but not others. pe38 in all Group I NPV and four GV genomes	Bind to host transcription factors	[61]
	Lef-6	All Lepidopteran NPVs and GVs	Lef-6 has a TAP (TIP associating domain), which can interact with nuclearporins for mRNA export to the cytoplasm	[6,62]
	Ac98-38 K protein	All baculoviruses	Predicted to have carboxyl terminal domain (CTD) phosphatase activities that negatively regulate RNA polymerase II by inhibiting RNA elongation	[6]
Translation arrest	P35, IAPs and P49	As mentioned before	Enhance early host translation arrest	[63]
	Protein kinase 2 (Pk-2)	PK2 found in AcMNPV, BmNPV, PlxyNPV and RoMNPV	Represses translation arrest, which is caused by host eIF2α kinase, by blocking eIF2α access to translation initiation factors	[64,65]
	Host range factor 1 (Hrf-1),	Only found in viruses of Lymantria dispar host, including LdMNPV and Orgyia pseudotsugata MNPV	Inhibits translational arrest by an unknown mechanism	[66]
	Hycu-ep32 gene	Hyphantri acunea NPV and OpMNPV	Induces host translation arrest by an unknown mechanism	[67]
	IAP-1 and IAP-2	As mentioned before	IAP1 and IAP2 possess ubiquitin ligase activities, enabling polyubiquitination of insect proteins, thus marking them for degradation	[68]
Growth & development	Protein tyrosine phosphatase (PTP)	All Lepidopteran Group I NPVs, not others, orthologs found in insect host	Induces host hyperactive behaviours	[69,70]
	Viral Fibroblast growth factor (vFGF)	All baculoviruses, orthologs found in insect hosts	Increases host larvae motility by facilitating systemic infection	[71,72]
	Chitinase and Cathepsin	Chitinase and Cathepsin in all Group I (except AgMNPV), all Group II (except AdhoNPV for Chitinase) and four GVs	Chitinase breaks larvae chitin layer, Cathepsin is a viral proteinase	[73]
	Ecdysteroid UDP glucosyl transferase (EGT)	All Lepidopteran Group I NPV, not others	Prevents moulting to extend insect life and virus propagation time (transfers glucose group to inactivate insect molting hormone ecdysteroids), induces host hyperactive behaviours	[74,75]
MicroRNA	BmNPV-miR-1	Conserved in AcMNPV, BomaNPV, PxMNPV, RoMNPV, and MaviNPV	Down-regulates the transport of host small-RNA from the nucleus to the cytoplasm, thereby reducing active population of host small RNAs	[76]
	BmNPV-miR-2 to 4	Conserved in AcMNPV, BomaNPV, PxMNPV, RoMNPV, and MaviNPV	Potentially targets 8 viral genes and 64 host genes	[77]

**Table 2:** Host genes that respond to/are affected by baculovirus infections*.

Functional groups	Insect genes/pathways	Functions	Virus-host systems and expression time	Ref.
Immune genes	Gloverin	An antibacterial and antiviral protein that interacts with the lipid envelope surrounding viral nucleocapsids	BmNPV-*B. mori* larvae and AcMNPV-*S. exigua* larvae (induced from 6–12 h.p.i)	[19,31,78]
	Cecropin	A cationic antimicrobial peptide that has positively charged regions in its α-helical peptide and interferes with the lipid membrane	*H. virescens* larvae-HzSNPV and HzAM1 cells-HearNPV(induced from 12 h.p.i)	[7,33,79]
	Apoptosis genes	Down regulation of host IAPs, and up-regulation of host apoptosis enhancers and Caspases trigger apoptosis upon virus infections	*Epiphyas postvittana* larvae-EppoNPV (5 d.p.i); HzAM1 cells-HearNPV (12 h.p.i); and Sf21-AcMNPV (before 24 h.p.i)	[15,18,80]
Signal transduction	Phosphatidylinositol 3 kinases (Pi3K)-Akt pathway	Elevation of this pathway prevents apoptosis and creates inductive environment for virus propagation	AcMNPV-Sf9 (induced from 1–18 h.p.i)	[81,82]
	MAPK pathways	Extracellular signal-regulated kinase (ERK) and c-Jun NH2-terminal kinase (JNK) pathways are activated at late infection and important for virus production	BmN4 cells-BmNPV (induced from 4–24 h.p.i)	[11,83]
	DNA damage response kinases	Triggered by virus replication‚ leading to cell death	AcMNPV-Sf21 (induced from 2–24 h.p.i)	[9]
Metabolic genes	ABC transporters and sugar transporters	ABC transporters transport a broad spectrum of substrates, including degradation products from cytosol to ER	BmNPV-*B. mori* cell line and AcMNPV-*S. exigua* larvae (induced from 2–12 h.p.i); HearNPV-HzAM1 (induced from 12–18 h.p.i)	[7,19,32,84]
	Citrate synthetase	Important for energy generation (a key enzyme in the Citric acid cycle, TCA)	AcMNPV-Sf9 and BmNPV-*B. mori* cell line (induced from 2 h.p.i)	[14,85]
	Pyruvate dehydrogenase/Aldehyde dehydrogenase	Important for energy generation and diversion of substrates to lipid biosynthesis	AcMNPV-Sf9 induced from (6 h.p.i)	[12]
	Lipid reductases and lipid desaturases	Fatty acid metabolism	HzNPV-*H. virescens (*induced from 12 h.p.i)	[33]
	Genes involved in cellular iron (iron ion transport, ferric iron binding, and cellular iron ion homeostasis)	Iron is important for processes such as DNA replication and ATP generation	BmNPV-*B. mori* cell line (induced from 3–6 h.p.i); *H. virescens* larvae-HzSNPV (induced from 24–72 h.p.i)	[11,86]
	Mitochondrial respiratory genes	Important for energy generation	BmNPV-*B. mori* cell line (induced from 1.5–24 h.p.i)	[7,11]
Translation	Heat shock protein (HSP) 70, HSP90, and Heat shock protein cognate (HSC) 70	Protein folding and facilitate several cellular processes conducive for virus replication such as ubiquitin-proteasome pathway	BmNPV-*B. mori* larvae (6–12 h.p.i); AcMNPV-Sf21 cell line (induced from 6–48 h.p.i);AcMNPV-*S. exigua* larvae (induced from 12 h.p.i)	[10,19,31,87]
	ER proteins (reduced)	ER stress	AcMNPV-Sf21 and AcMNPV-Sf9 (induced from 12–48 h.p.i)	[10,12]
	Translation initiation factors (TIFs)	Enhance translation	BmNPV-*B. mori* cell line (induced from 1.5–24 h.p.i)	[11,19]
	eIF2α	Phosphorylation of eIF2α causes translation arrest	AcMNPV-Sf9 cell line (Before 36 h.p.i)	[64,88]
Replication	Histone genes	Regulate host chromatin structure, which affects DNA replication	BmNPV-*B. mori* cell line (induced from 1.5–24 h.p.i)	[11]
Transcription	Host’s polyhedrin promoter binding protein (PPBP)	Binds to promoters of both p10 and polyhedrin genes to enhance their transcription	AcMNPV-Sf9 (induced late)	[89]
	Transcription initiation factors	Enhance transcription	BmNPV-*B. mori* cell line (induced from 6–24 h.p.i); HearNPV-*H.zea* (induced from 18 h.p.i)	[7,11]
mRNA and protein degradation	Alkaline nuclease	mRNA degradation	E. postvittana larvae-EppoNPV (induced from 5 d.p.i); BmNPV-B. mori cell line (induced from 6 h.p.i)	[15,32]
	Ubiquitin-proteasome pathway	Protein degradation	BmNPV-*B. mori* cell line (induced from 2–6 h.p.i); Sf9-AcMNPV (induced from 1.5–48 h.p.i)	[ 11,14,90]
Cytoskeleton	Dynein	A motor protein involved in microtubule transport	BmNPV-*B. mori* cell line (induced from 6–12 h.p.i)	[32]
Development	Juvenile hormones	Maintaining juvenile hormones at high level extends growth and inhibits moulting	*H. virescens* larvae-HzSNPV (induced from 12 h.p.i); *E. postvittana* larvae*-EppoNPV* (induced from 5 d.p.i)	[15,33]
MicroRNAs	miRNA (90 miRNAs in Sf, 114 in *B.mori*)	Play roles in antiviral response by degrading viral transcripts (e.g. bmo-miR-8 potentially targets IE1)	Sf9-AcMNPV (Usually induced at late infection, 24–72 h.p.i)	[13,91]
	Dicer 2	Produce viral short interfering siRNAs that degrade viral transcripts	*H. armigera* larvae—HearNPV (induced from 48 h.p.i)	[92]
Transposition of host DNA	Host transposable elements (retrotransposons), reverse transcriptase, gag/pol-like proteins	DNA transposition into baculovirus genomes, contributing to virus genome instability.	Sf9 cells, *T.ni* cells, *H. virescens* larvae-HzSNPV and AcMNPV-*S. exigua* larvae (During *in vitro* serial passaging)	[19,33,93,94]
Detoxification	Glutathione S-transferase (GST)	Convert glutathione into water-soluble, less toxic metabolites	AcMNPV-*S. exigua* larvae (induced from 12 h.p.i)	[19]

^*^ In contrast to Table 1‚ which shows mostly findings from molecular studies based on the analysis of virus genes‚ this Table shows that a majority of current understanding on insect responses to baculovirus infections have been obtained from genome scale transcriptomic expression studies (those highlighted in bold and red text).

### 2.2. Metabolism

In relation to energy, no gene for an enzyme involved in energy metabolism has been found in any sequenced baculoviruses. However, a number of processes during virus infection require a high energy supply. For example, DNA packaging into nucleocapsids requires Adenosine triphosphate (ATP) for motor protein activities [95], elongation during translation uses Guanosine triphosphate (GTP) energy for binding of tRNA onto ribosomes, and active substrate transport, such as that provided by ABC transporters, the uptake of amino acids into cells, the export of viral RNA and the import of viral proteins cross the nuclear membrane, plus the directional transport of nucleocapsids all consume extensive energy [44]. Therefore, baculoviruses rely on host enzymes for energy metabolism. Transcriptomic studies show that ABC transporters and Citrate synthetase increased in Sf9 cells infected by AcMNPV [32,84]. These studies also identified an increase in the level of Glyceryldehyde 3-phosphate dehydrogenase (GAPDH) in HzAM1 cells upon infection by HearNPV [7], and in other hosts infected by DNA viruses‚ including Vaccinia virus infected human monocytes [96] and Rock bream Iridovirus infected marine teleost cells [97]. Similarly, up-regulation of Aldehyde dehydrogenase was found in both *S. exigua* cells infected by AcMNPV [19] and AcMNPV infected Sf9 cells [12]. Importantly, how the virus manipulates host energy generation and metabolic pathways to foster virus replication is still largely unknown.

For synthesis of virus building blocks such as nucleotides and amino acids, there are known virus genes for nucleotide metabolism, encoding a Ribonucleotide reductase (RR) large subunit and a small subunit, and a dUTPase protein, which prevents incorporation of mutagenic dUTP into DNA, suggesting that nucleotide metabolism may be especially important for baculovirus infections [57,58]. Via transcriptomics, Breitenbach *et al.* [33] found an increase in fatty acid metabolism in infected insects, including genes encoding lipid reductases and lipid desaturases in *H. virescens* larvae infected by HzSNPV. However, an extensive range of responses by metabolic genes have not been identified in baculovirus infected cells, especially those related to nucleotide and amino acid metabolism, which are important for production of new viruses in infected cells. 

### 2.3. Translation

For translation, baculoviruses are entirely dependent on the host translational machinery including ribosomes, tRNAs, amino acid metabolism and transport, chaperones for protein folding, endoplasmic reticulum (ER) for glycosylation/phosphorylation/transport, and other translation factors. Several mechanisms allowing baculovirus mRNAs to compete with insect host mRNAs for translation have been found. These include the presence of AT-rich regions and unstructured 5' untranslated (UTR) regions [8,98], which confer a higher binding affinity for host translation factors by virus mRNAs compared to that of host mRNAs; the relative independence of virus late mRNAs on the 5'-cap-binding eukaryotic initiation factor (eIF4E) for the ribosomal recruitment process, in contrast to most insect mRNAs, which are dependent on cap-binding proteins for being translated [98,99]; and the ability of several virus genes, namely protein kinase 2 (pk-2) and host range factor 1 (hrf-1), to inhibit translational shutoff [64,66]. Emerging evidence also suggests that baculoviruses up-regulate translation initiation factors to accelerate the translation process [7,11]. 

Up-regulation of host genes for protein folding, translation initiation, and translational arrest have been reported. Like many other viruses, baculoviruses utilize host chaperones to facilitate the rapid synthesis of a large amount of viral proteins [87]. Heat shock proteins (HSPs) and heat shock protein cognates (HSCs) were commonly up-regulated in AcMNPV-*S. frugiperda*, HearNPV-*H. zea*, HzSNPV*-H. virescens*, *B. mori*-BmNPV, and *S. exigua*-AcMNPV infections [10,18,19,32]. Similarly, the up-regulation of other translation factors was commonly found, including: eukaryotic initiation factors (eIF3-6, eIF1A, eIF3-2b) and an elongation factor (EF1d) in *B. mori*-BmNPV infections [11]; eIF in *S. exigua*-AcMNPV infections [19]; and IF-2 and IF-3 in *H. zea*-HearNPV infections [7]. In addition, baculovirus infections trigger phosphorylation of the Eukaryotic initiation factor (eIF2α), which potentially causes translation arrest, but the host protein synthesis machinery is still functional at the late infection stage, as shown by detection of polysomal translation activities [16]. Protein degradation is one of the major responses of the host cell to a baculovirus infection. Proteasome 26S subunit and the ubiquitin-proteosomal degradation pathway in general were up-regulated in silkworm cells infected by BmNPV [11,32]. There is also evidence for baculovirus infections causing Endoplasmic reticulum (ER) stress [33]. More research is needed to better understand the roles of the ER, ribosomes, and translation factors needed for facilitating viral protein production.

### 2.4. Transport

For transport processes in infected cells, nucleocapsid movement is relatively well understood, while there is less knowledge on transport of viral proteins for virus DNA packaging, nucleocapsid assembly, and occlusion processes. Baculoviruses have devised effective transport mechanisms that are dependent on host Actin and cause a cytoskeleton remodeling phenomenon, in which both microfilaments and microtubules are used [44,45,46,49,100]. Nucleocapsid transport was detected within the cytoplasm as early as 5–30 min post infection, and inside the nucleus within one hour post infection [45]. A number of virus genes have been found to be involved in the nucleocapsid transport process, including: VP80, P78/83, EXONO, p10, VP39, ODV-c42, Ac66 and Arif-1 (Actin rearrangement inducing factor) [46]. For assembly, traditional protein techniques using yeast-two-hybrid screening for studying ODV protein-protein interactions have identified five triple-interactions, eight double- and nine self-interactions among virus ODV proteins [101]. Interactions of virus proteins with nuclear actin filaments and microtubule systems are essential for nucleocapsid assembly [44,45]. For transport of ODV proteins from the ER to the inner nuclear membrane, the host Importin-α-16, the viral FP25K (interacts with all known ODV proteins) and the ODV-E26 protein (interacts with some ODV proteins) play important roles [51]. The nuclear importins and exportins are also important for exporting viral mRNA to the cytoplasm, but their specific roles for transporting viral mRNA have not been investigated. BV envelope proteins are *N*-glycosylated and are transported from the ER to the cellular membrane, where nucleocapsids bud out. DNA packaging involves a packaging motor complex, possibly contains a virus Ac66 protein which interacts with host myosin and ATPase, and likely involves host actin and microtubules [6]. The occlusion process uses micro vesicles derived from the inner nuclear membrane [102]. Besides temporal differences in relation to BV *versus* ODV formation, it is unknown how virus genomes are directed to BV assembly rather than to the ODV occlusion process. The finding that a number of host proteins are incorporated into BV structures indicates that a number of unknown host proteins may be important for virus assembly, or for BV functions when entering new cells [11]. Thus, although the movement and remodeling of the host cytoskeleton system has been well studied, the roles of host genes in viral mRNA and viral protein transport and in virion assembly are still poorly understood.

### 2.5. Replication

For virus replication, baculoviruses have evolved a near complete replication machinery, which concentrates in a separate compartment within the nucleus, called the virogenic stroma [103]. Known replication enzymes that baculoviruses possess include: DNA polymerase (capable of both leading and lagging strand synthesis as well as proof reading by the 3'–5' exonuclease activities), helicase (p143), late expression factor LEF-1 (late expression factor, primase), LEF-2 (primase accessory factor), LEF-3 (single stranded DNA binding protein), p143 (helicase), origin binding protein IE1 (immediate early), and origin of replication with homologous regions (hrs) and non-homologous regions (non-hrs) (reviewed by Rohrmann [6] and Vanarsdall *et al.* [59]). Compared to virus transcription, replication may require more host factors, such as host DNA topoimerases (to untwist the double stranded DNA, thereby facilitating helicase activities) and DNA ligases (to link Okazaki fragments at the lagging strand). However, the host DNA polymerase is unable to substitute the viral DNA polymerase to complete virus DNA replication [103]. On the other hand, a baculovirus DNA polymerase can be interchanged with that from another baculovirus or even with a distantly related ascovirus DNA polymerase [104], suggesting that there is flexibility for baculovirus DNA polymerase activities, and hence it is possible that host DNA replication factors can facilitate viral replication. It remains unknown which host replication factors are used for virus replication, an efficient process that eventually leads to over 20 times more virus DNA than total host DNA in an infected cell [7].

For host DNA replication, cellular DNA replication is arrested, but viruses can utilize the host replication machinery to facilitate viral replication. Under baculovirus infection conditions, while cell cycle progression is abandoned at the G1/S or G2/M checkpoint (by 4 h.p.i, or 10 h.p.i in AcMNPV and 24 h.p.i for HearNPV systems), virus DNA replication increased rapidly [105]. Baculovirus replication, by a rolling cycle and recombination process, causes a cellular DNA damage response (DDR) following phosphorylation of several Phosphoinositide 3-kinases (Pi3K), which phosphorylate targeted substrates in the nucleus, for example the Histone 2A variant, and eventually causes cell cycle arrest or apoptosis [9]. The host DDR is a conserved response found by many host cells following infection by DNA viruses [106]. The up-regulation of Histone 2A variants was also found in *B. mori* cells infected by BmNPV [11]. Interestingly, even though the DDR is considered as a defence response by the cells, baculoviruses require this process for promoting virus replication and inhibition of DDR reduced virus production by 100,000 times [9]. Cellular replication factors, such as DNA polymerase subunits, could also be utilized by the virus for viral replication [7]. Knowing how the virus makes full use of the cellular replication machinery is important for maximizing virus production.

### 2.6. Transcription

For virus late transcription, baculoviruses possess a complete and effective transcription system to express viral genes under stringent temporal patterns. The viruses have evolved a unique RNA polymerase complex that can transcribe genes and process mRNAs at both ends by capping and polyadenylation. The complex contains 4 subunits‚ which are: LEF-4, an RNA capping enzyme; LEF-8, similar to the large subunit β in Bacteria; LEF-9, similar to the large subunit β’ in Eukaryotes; and p147 similar to the alpha subunit in Bacteria. Virally encoded transcription enhancers include: a very late factor VLF-1‚ which binds to a burst sequence to make very late promoters accessible to RNA polymerase; a LEF-5 gene as a transcription initiation factor; and different DNA sequence motives within early and late promoters. Virus genes for mRNA processing include: the LEF-4 and an RNA cap 2’*O*-methyltransferase that are responsible for capping by RNA triphosphatase and Guanine transferase activities; an ADP-ribose pyrophosphatase for decapping; and a gene for polyadenylation‚ which is the viral RNA polymerase II with a distinct mechanism different to the host’s polyadenylation process [107]. The viruses also encode a putative mRNA exporter (LEF-6). Nevertheless, the host factors are essential for immediate early transcription, when the virus transcription machinery has not been established. In addition, it is likely that the viruses also utilize host factors to facilitate its late transcription processes, such as a polyhedrin promoter binding protein (PPBP), which binds to promoters of both p10 and polyhedrin genes to enhance their transcription [89]. Xue *et al.* [11] predicted that all five virus proteins, including protein kinases (PK-1 and PK-2), CG-30, IE-2 and PE38, which display the most extensive interactions with over 1,000 insect genes, are related to transcription regulation, indicating that a high number of host proteins may contribute to virus transcription. Up-regulation of host transcription factors to facilitate the viral infection process deserves to be explored further. 

### 2.7. Immune responses

For apoptosis and cell cycle arrest‚ it is well known that baculoviruses have evolved anti-apoptosis genes (virus inhibitor of appoptosis IAPs, p25, and p49) to inhibit cell death responses and they carry a cell cycle arrest gene, a Cyclin homolog, to block G2/M progression and create a “pseudo S” phase conducive for virus replication. On the other hand, there is little research on virus infection and host signaling pathways to identify cellular modulators and mediators involved in apoptosis and translation arrest [108]. There is evidence that the virus DNA replication process via a rolling cycle and recombination mechanism trigger a host DNA damage response, via the P3i-Akt pathway, which leads to an apoptotic response. The virus anti-apoptosis counter-defense is by direct binding of virus anti-apoptotic proteins to host caspases or by triggering proteasomal degradation of caspases. It has been found for AcMPNV and BmNPV that one of the mechanisms for the viruses to trigger cell cycle arrest at G2/M phase is by a virus Cyclin, a homolog of the host Cylin-B, which stably binds to the host cell division cycle (cdc-2) protein, leading to prolonged cdc-2 activities and preventing cell cycle progression to anaphase [41,109]. 

Over-expression of antimicrobial peptides and apoptosis are common host defense responses found in insect-baculovirus systems. Gloverin, encoding an antiviral peptide‚ increased in BmNPV infected *B. mori* and in *S. exigua* larvae infected by AcMPNV [19,31]. Another antiviral peptide, Cecropin, is also commonly found up-regulated, such as in *H. virescens* infected by *Helicoverpa zea* single nucleopolyhedrovirus (HzSNPV) [33] and in the *H. zea*-HearNPV system [7]. Similarly, induction of apoptosis by baculovirus infections has been found in many expression studies using baculovirus-insect systems [10,11,18].

Recently‚ microRNA (miRNA) and small interfering RNA (siRNA) responses have been identified as important control mechanisms for insect-virus interactions. Rapid discovery of miRNAs is aided by the development of NGS technologies and computational analysis followed by validation of selected putative miRNAs. For baculoviruses, only four viral miRNAs have been detected by using NGS to sequence small RNAs extracted from BmNPV-infected *B. mori* cells [77]. These four miRNAs are evolutionarily conserved among many baculoviruses. On the other hand, many insect miRNAs, from 90–62 miRNAs, have been discovered. Using Illumina NGS, over 90 host insect miRNAs were found in *S. frugiperda* [13]. By sequencing 6,720 clones of small RNAs in *B. mori*, Yu *et al.* [91] found 35 miRNAs, while the computational prediction identified 262 genes for miRNAs. Comparing outputs by the two approaches, NGS generated more miRNAs than traditional sequencing. For siRNA, NGS also discovered siRNA responses in the *H. armigera* insect [92]. The insect cells encode a Dicer 2, which cuts double stranded viral dsRNA to generate viral small interfering siRNAs that target viral mRNA products during late infection periods [92]. In addition, some virus miRNAs may regulate host miRNA populations by inhibiting nuclear export of host miRNAs [76]. 

In relation to signaling pathways, the BmNPV virus induced the *B. mori* mitogen protein kinase (MAPK) pathways, including extracellular signal-regulated kinase (ERK), and c-Jun NH_2_-terminal kinase activity (JNK), possibly via the virus PK-1 protein [83]. AcMNPV infection increased Akt phosphorylation from 2–6 h.p.i and activated the Pi3-Akt pathway in infected cells [81]. The MAPK and Akt pathways are pro-survival pathways, often being activated before cells commit to apoptosis. Sagisaka *et al.* [32] found up-regulation of a Toll protein in *B. mori* cells infected by BmNPV (BmToll10-3, a surface protein receptor), but a change in the Toll pathway was not identified. Similarly, there have been no findings of changes to the Janus kinase (JAK)-signal transducer and activator of transcription (STAT) (JAK-STAT) pathway or the immune deficiency (IMD) pathway in baculovirus infected insect cells to date. Therefore how baculoviruses control, and insect cells respond, via signaling pathways‚ especially those involved in innate immune responses‚ remain to be explored.

## 3. Manipulation of Host Cells or Larvae to Study Baculovirus-Insect Interactions

Common methods applied for studying pathways in insects use chemical inhibitors to moderate a pathway, for example: using 5-fluoro-29deoxyuridine (FdUrd) for cell cycle arrest at G1/S [105], using U0126 and PD98059 for moderation of Extracellular signal regulated kinases (ERK) [69], using SP600125 for moderation of the JNK pathway [69], and using PS-341 for proteasome modifications [11]. Similarly; to trigger a pathway, Actinocyn D is used for inducing apoptosis [110]. Molecular biology methods for testing roles of a pathway or a gene to baculovirus infection in insects most commonly use gene knockdown by RNA-interfering (RNAi) technology, for example using RNAi to repress the MAPK pathway (JNK and ERK) [69] or apoptotic caspases [111]. Until recently, RNAi silencing technology has been used successfully for studies involving 15 Lepidopteran species [112]. A technique for targeted gene knockout, using Zinc Finger Nuclease technology, was also successfully applied for studies with *B. mori* [113]. For over-expressing a gene or introducing a heterologous gene into insect cells, transient transfections have been widely applied with a range of transfection reagents, including inexpensive and effective reagents such as polyethylenimine (PEI) [114]. Tools are also available for stable transfection, most commonly using antibiotics for selecting cells containing heterologous DNA randomly inserted into insect cell chromosomes [115]. More details about methods for genetic engineering of the baculovirus-insect system are reviewed elsewhere [116,117]. 

## 4. Conclusions and Future Perspectives

Overall, while baculoviruses have evolved to control insect hosts‚ there is increasing evidence of host cell responses against baculovirus infections. Figure 1 summarizes many of the bidirectional interactions between cell and baculovirus genes/proteins that have been discussed in this review. However, host responses are relatively poorly understood compared to current knowledge on virus genes. Metabolic pathways including energy and nucleotide/amino acid/lipid metabolism are expected to be up-regulated‚ but little is known about which genes, if any, are affected. Likewise‚ how the viruses control various pathways such as signaling pathways are still largely unanswered questions. Similarly‚ although heat shock responses are now known to be a conserved mechanism beneficial to baculoviruses and have been found in all studied baculovirus-insect cell systems, the trigger of the response by these host genes to baculovirus infections remains unknown. 

“Omics” tools can greatly improve the knowledge landscape of baculovirus-insect interactions by investigating cellular responses at a genome scale. It can be seen from Figure 1 that the right half of the diagram represents cellular pathways rather than virus-related processes, indicating a lack of research on cellular processes affected by virus infections. This lack of understanding of cellular responses is mainly due to the unavailability of genomic sequences as well as the longer time required to engineer an insect genome than the time required to edit a virus genome. To date, RNA-seq technology is revolutionizing research in organisms without genomic sequences by generating transcript sequences and allowing the analysis of expression levels of these transcripts [118,119]. Transcriptomic approaches enable more comprehensive studies, which are at a whole genome scale (involving over 15,000 genes), generally far more genes compared to the number of proteins or metabolites being measured by proteomic or metabolomic approaches.

A number of studies comparing microarray and RNA-sequencing (RNA-seq) have shown that the two tools produce highly correlated results (r > 0.91) and they complement each other [119,120]. RNA-seq has advantages over microarrays in the discovery of novel transcripts, analysis of isoforms, and detection of antisense, splice junctions, siRNA, and miRNA. However, for non-model organisms that do not have reference genomes, accurately assembling transcripts and mapping of reads onto transcripts are fundamentally important for determining differential expression results using RNA-seq [119]. In addition, it is speculated that RNA-seq might produce bias for highly expressed virus genes at late infection stages when the polyhedrin and p10 genes are hyper-expressed, and detection of low abundant genes could be compromised [11]. Combining results from microarray and RNA-seq analysis can increase detection power and accuracy [120].

Compared to transcriptomics, proteomics and metabolomics are in an early stage of development, but rapid progress is being made with these tools. Proteomics with mass spectroscopy based approaches and activity-based functional validation procedures can provide insights into host-virus interactions [121]. A proteomic study of infections in permissive, semi-permissive and non-permissive cell lines, *H. virescens* and *H. zea*, by AcMNPV and HzSNPV identified 21 proteins, among which the up-regulation of two Calreticulin proteins, two DNA supercoiling factors, and two heat shock proteins by 5-fold were found [122]. Courtiade *et al.* [123] carried out a proteomic study using *H. armigera* cells following induction of apoptosis and identified changes in 13 proteins compared to control cells. Chen *et al.* [124] found nine proteins being changed in susceptible and resistant *B. mori* midgut tissues following virus infections. The current metabolomics technology only allows measurements of a part of the metabolome [125]. Among a few metabolic studies in insects so far, measurements of amino acids, sugars and several redox agents have been undertaken. Tran *et al.* [126] measured amino acids and adenosine phosphates Bernal *et al.* [127] measured glucose, glutamine, glutamate, lactate, ammonia, maltose, sucrose, glucose, NAD^+^/NADH and performed assays for 10 enzyme activities. At this stage, the rapid development of quantitative proteomics allows identification and measurements of thousands of proteins [128]. Targeted metabolomics is also powerful in unraveling changes in host metabolism [129], an area that remains largely unexplored for baculovirus-insect systems.

Engineering insect cells for functional analysis of target genes in insect-virus interactions can use high throughput screening technologies, involving the use of RNAi systems [130]. Although no high throughput gene knocking technologies have been established to date in insect-baculovirus systems, temporary expression by transient transfection of expression vectors or creating stably transfected polyclonal cells can be good options to validate genes that require up-regulation or over-expression to improve production outcomes using insect cell technology. These technologies together with the power of “omics” tools, especially the two well established transcriptomic tools, can unravel many host pathways that are yet to be fully explored.
